# Esketamine Treatment Trajectory of Patients with Treatment-Resistant Depression in the Mid and Long-Term Run: Data from REAL-ESK Study Group

**DOI:** 10.2174/011570159X337670241029062524

**Published:** 2024-12-31

**Authors:** Gianluca Rosso, Giacomo d’Andrea, Stefano Barlati, Marco Di Nicola, Ileana Andriola, Matteo Marcatili, Vassilis Martiadis, Miriam Olivola, Stefania Di Mauro, Gabriele Di Salvo, Pasquale De Fazio, Massimo Clerici, Bernardo Maria Dell’Osso, Antonio Vita, Giorgio Di Lorenzo, Mauro Pettorruso, Giovanni Martinotti, Giuseppe Maina

**Affiliations:** 1 Department of Neurosciences ‘Rita Levi Montalcini’, University of Torino, Turin, Italy;; 2 Psychiatric Unit, San Luigi Gonzaga University Hospital, Orbassano, Turin, Italy;; 3 Department of Neurosciences, Imaging and Clinical Sciences, Università degli Studi G. D'Annunzio, Chieti, Italy;; 4 Department of Mental Health, ASL 2 Abruzzo Lanciano-Vasto-Chieti, Chieti, Italy;; 5 Department of Clinical and Experimental Sciences, University of Brescia, Brescia, Italy;; 6 Department of Mental Health and Addiction Services, ASST Spedali Civili of Brescia, Brescia, Italy;; 7 Department of Neurosciences, Section of Psychiatry, Università Cattolica del Sacro Cuore, Rome, Italy;; 8 Department of Psychiatry, Fondazione Policlinico Universitario “Agostino Gemelli” IRCCS, Rome, Italy;; 9 Department of Translational Biomedicine and Neuroscience, Università degli Studi di Bari Aldo Moro, Aldo Moro, Italy;; 10 Department of Mental Health and Addiction, Fondazione IRCCS San Gerardo dei Tintori, Monza, Aldo Moro, Italy;; 11 ASL Napoli 1 Centro, Department of Mental Health, Napoli, Italy;; 12 Department of Brain and Behavioural Science, University of Pavia, Pavia, Italy;; 13 Department of Mental Health and Addictions, Azienda Socio-Sanitaria Territoriale (ASST), Pavia, Italy;; 14 SPDC Frosinone - ASL Frosinone, Frosinone, Italy;; 15 Psychiatry Unit, Department of Health Sciences, University Magna Graecia of Catanzaro, Catanzaro, Italy;; 16 School of Medicine and Surgery, University of Milano-Bicocca, Monza, Italy;; 17 Department of Biomedical and Clinical Sciences Luigi Sacco, Aldo Ravelli Center for Neurotechnology and Brain Therapeutic, University of Milan, Milano, Italy;; 18 Chair of Psychiatry, Department of Systems Medicine, University of Rome Tor Vergata, Rome, Italy;; 19 IRCCS Fondazione Santa Lucia, Rome, Italy;; 20 Institute for Advanced Biomedical Technologies, ITAB, “G. d'Annunzio” University of Chieti-Pescara, Chieti, Italy;; 21 Psychopharmacology, Drug Misuse and Novel Psychoactive Substances Research Unit, School of Life and Medical Sciences, University of Hertfordshire, Hatfield AL10 9AB, UK

**Keywords:** Major depressive disorder, treatment-resistant depression, antidepressants, esketamine, maintenance treatment, treatment outcome

## Abstract

**Introduction/Objective:**

Data on long-term treatment with Esketamine Nasal Spray (ESK-NS) in real-world patients with treatment resistant depression (TRD) is scarce. The primary aim of the study is to evaluate the effectiveness and tolerability of ESK-NS treatment at 6 and 12-month follow-ups.

**Methods:**

This is part of an observational, retrospective, multicentric Italian study (REAL-ESK study). Subjects for the present study underwent psychiatric assessments after 6 and 12 months from the start of ESK-NS treatment. Repeated measures analysis of variance (ANOVA) was used to assess changes in continuous variables, such as scores on psychometric scales from baseline to follow-up time points.

**Results:**

Of 63 patients who maintained ESK-NS treatment for at least 6 months, 48 were responders or remitters (76.2%). Among 15 non-responders at 6 months, 4 significantly improved at 12-month follow-up. At least one side effect was reported by 71.8% of subjects with a 6-month follow-up assessment. An overall reduction of side effects was noticed as treatment progressed (42% of patients who continued the treatment reported side effects at 12 months). The most common side effects were sedation (31.7%) and dissociation (28.6%) during ESK-NS sessions. Only 2 patients discontinued ESK-NS for tolerability reasons.

**Conclusion:**

The results support the effectiveness and safety of esketamine in the mid and long-term treatment of TRD patients. The late clinical response of a subgroup of patients represents a novel finding. Data needs to be confirmed in larger samples and longer observation periods.

## 1 INTRODUCTION

The most commonly used operational definition of treatment resistant depression (TRD) is a major depressive episode (MDE) that fails to respond to at least two different antidepressants (ADs) given at adequate dose and duration [1]. According to this definition, approximately 30-50% of patients with major depressive disorder (MDD) have a TRD [2]. The naturalistic treatment patterns and clinical outcomes of real-world TRD patients in Europe during the period 2018-2020 were characterized by high rates of no response to a new treatment strategy both at 6 and 12 months (73.5 and 69.2%, respectively) and substantial periods of persistence in the same treatment despite high levels of non-response [3, 4]. These data supported the existence of an unmet treatment need for TRD patients in Europe, with considerable healthcare resource utilization and associated economic impact [5].

Within this framework, Esketamine Nasal Spray (ESK-NS) has been introduced in the market as a new pharmacological option for TRD. A phase 3 short-term trial of ESK-NS plus an oral AD (SSRI or SNRI) demonstrated a significant reduction in depressive symptoms in TRD patients compared with an oral AD plus placebo nasal spray at 4 weeks [6]. ESK-NS has also been compared to quetiapine extended-release, both added to an SSRI or SNRI, in an open-label single-blind, randomized, active-controlled trial, with favorable outcomes for ESK-NS both at week 8 and over 32 weeks of follow-up [7]. Moreover, observational studies have addressed the question of the effectiveness and tolerability of ESK-NS in real-world TRD patients, showing data consistent with registration trials and adding new insight on the gradual increase of response/remission rates until 2-3 months from the start of ESK-NS treatment [8, 9]. Regarding the maintenance treatment, according to the summary of product characteristics, ESK-NS plus an oral AD should be continued at least 6 months after a significant improvement of depressive symptoms. An RCT showed the risk of relapse/recurrence to be lower in patients who continued with ESK-NS for one year after response/remission in comparison to those switched to placebo nasal spray; particularly, the risk of relapse/recurrence in case of discontinuation of ESK-NS appeared to be lower in remitters than responders [10]. Today, data on long-term effectiveness and tolerability of ESK-NS in real-world TRD patients is scarce, and little is known about treatment persistency and potential predictors of relapse/recurrence.

In light of the above, the primary aim of the present study is to evaluate the effectiveness and tolerability of 6- and 12-month treatment with ESK-NS in real-world TRD patients; the secondary aim is to compare the characteristics of patients who continued ESK-NS treatment in the maintenance phase and those who did not.

## 2 MATERIALS AND METHODS

### 2.1 Participants and Study Design

Data presented are part of the REAL-ESK study, an observational, retrospective, multicentric study of subjects with TRD treated with ESK-NS [9]. The study was approved by the local ethics committee of the University of Brescia (Protocol Number: NP5331).

All REAL-ESK patients fulfilled the following criteria: a) age ≥ 18 years; b) diagnosis of MDE as part of a MDD; c) failure - in the current MDE - of at least two prior AD treatments at adequate doses, duration, and adherence, according to TRD consensus criteria [11]; d) ongoing treatment with at least one SSRI or SNRI according to ESK-NS summary of product characteristics, e) combination with other antidepressant and augmentation strategies with mood stabilizers and/or antipsychotics were also allowed. Exclusion criteria included: a) diagnosis of bipolar disorder or psychotic disorders; b) comorbid medical diseases, such as untreated hypertension or prior cerebrovascular disorders, which contraindicate ESK-NS administration. All patients were treated with ESK-NS in compliance with the indications provided by the Italian regulatory agency for drugs (Agenzia Italiana del Farmaco; AIFA) and the common clinical practice of TRD management.

Indeed, the maintenance phase was started in all those patients who showed at least an initial improvement in depressive symptoms during the first months of treatment. As for the continuation to 12 months, treatment was extended in patients who showed at least a partial response even if they did not fulfill the criteria for symptomatic full response or remission.

Many of the Italian mental health facilities involved in the REAL-ESK study provided the follow-up data analyzed in the study: the coordination centers being both the ‘G. d'Annunzio’ University of Chieti and the University of Brescia. Other centers involved were Fondazione Policlinico Universitario Agostino Gemelli IRCCS of Rome, ‘A. Moro’ University of Bari, University of Rome Tor Vergata, ‘Milano Statale’ University, ‘Milano Bicocca’ University, ‘Magna Graecia’ University of Catanzaro, University of Pavia, University of Torino, ASL Frosinone, ASL Napoli 1, ASP Messina.

### 2.2 Study Procedures and Measurements

Anamnestic data were retrospectively collected and included information on sociodemographic factors, the history of the disease, the treatment history for the current depressive episode, antidepressant trials experienced during the current episode, augmentation strategies (combined use of mood stabilizers/antipsychotic medications or not) and other therapeutic tools applied to treat TRD.

Psychometric assessments at T0, after six months (T1) and 12 months (T2) from the start of treatment, have been taken into consideration for the aims of the present study. Data were also collected in cases of premature study withdrawal or the occurrence of clinically relevant events, such as admission to or discharge from inpatient care, symptom relapse, or remission of major depressive episodes.

The Montgomery-Asberg Depression Rating Scale (MADRS) was used by clinicians to characterize depressive symptom. Patients were defined as responders with an overall 50% reduction in the MADRS score compared to the baseline assessment, while remission was defined as a MADRS score <10. The Hamilton Anxiety Rating Scale (HAM-A) was used to assess the severity of anxiety symptoms and their reduction during treatment.

### 2.3 Statistical Analyses

Statistical analyses were performed using SPSS 28.0 software (SPSS Inc., Chicago, IL, USA). All tests were two-tailed, with a statistical significance level set at *p* < 0.05. Continuous variables are expressed as mean ± standard deviation (SD), while categorical variables are reported as average numbers and percentages. Given a significance level of 0.05, the post hoc power analysis on the sample size of 78 patients showed a statistical power > 95% related to MADRS mean scores. Since data followed a normal distribution at baseline (Kolmogorov-Smirnov test: 0.08; p: 0.20), parametric tests were used. Repeated measures analysis of variance (ANOVA) was used to assess changes in continuous variables, such as psychometric scales, from baseline (T0) to follow-up (T1 and T2), whereas the Pearson χ^2^ test was performed for categorical variables. Comparison analyses at 6-month follow-up (T1) were conducted between patients who stopped or continued ESK-NS and between responders and non-responders.

## 3 RESULTS

The socio-demographic and clinical characteristics of the 78 subjects included in the study are shown in Table **1**: the mean age was 51.8 (± 12.6) years, 55.1% were women, 53.8% were employed; for what concerns clinical history, the mean duration of the current MDE at the start of ESK-NS treatment was 15.6 (± 11.5), and the majority of patients suffered from recurrent MDD, with 5 previous MDEs average.

Thirty-seven patients (37.4%) had psychiatric comorbidities. In particular, 11 patients (29.7%) had an anxiety disorder, 8 patients (21.7%) had a personality disorder, 6 patients suffered from OCD (16.2%) and PTSD (16.2%) respectively, 3 patients (8.1%) had a previous history of substance use disorders and the remaining three patients suffered from anorexia nervosa (2.7%), gender dysphoria (2.7%) and autism spectrum disorder (2.7%).

The majority of patients underwent and failed 3 or more previous adequate AD treatments during the current MDE and many had ongoing pharmacological treatment with combinations of ADs (46.25%) and/or antipsychotics as augmentation’s agents (46.2%) and/or mood stabilizers (57.7%), beyond an SSRI or SNRI agent according to ESK-NS treatment guidelines. In particular, regarding the combination strategy with other antidepressants, 20 patients were treated with mirtazapine (55.6%) and 16 with bupropion (44.4%). As for the augmentation strategies with antipsychotics, 25 patients were taking quetiapine (69.4%) and 11 olanzapine (30.6%), while regarding mood stabilizers, 32 patients were receiving lithium in add-on (71.1%), 6 lamotrigine (13.3%) and 7 pregabalin (15.6%).

**Fig. (1):**
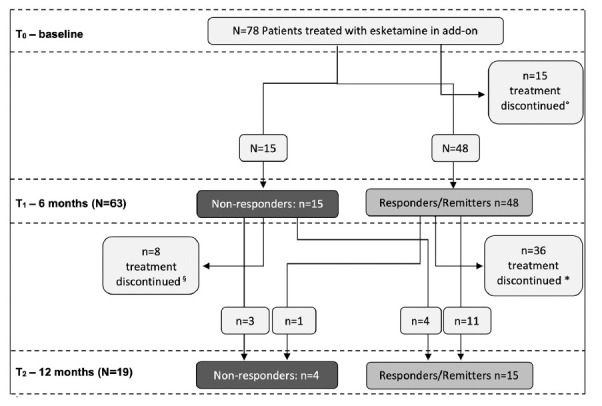
Patients in long term treatment with esketamine at each assessment point. °n.1 discontinued for clinical effectiveness, ^§^n.2 discontinued for clinical effectiveness, *n.26 discontinued for clinical effectiveness.

Of 78 patients included, 63 continued ESK-NS treatment for at least 6 months (Fig. **1**). Of those, 48 were responders or remitters (76.2%) and 15 (23.8%) were non-responders. Fifteen patients stopped ESK-NS before the 6-month time point: the reasons for the discontinuation are shown in Table **2a**: the major causes were low effectiveness and organizing problems such as medication supply or logistics of the centers involved in the study. Only in one case, esketamine was discontinued due to clinical remission before six months.

Forty-four discontinued ESK-NS between 6- and 12-month time points and the main reason was ‘clinical response’ as shown in Table **2b**. Of 19 patients that continued ESK-NS treatment until 12-month time point, 15 (78.9%) were responders/remitters and 4 (21.1%) were non-responders. Among the 15 responders/remitters at 12-month follow-up, 4 came from the non-responders group at 6-month. Putting together the 15 responders/remitters that continued ESK-NS until 12-month time point and the 28 that stopped ESK-NS due to clinical improvement after at least 6 months of treatment, a total of 43 patients on 78 (55.1%) had significant benefit from maintenance treatment with ESK-NS.

Fig. (**2**) shows the MADRS mean variations of the patients who continued ESK-NS until 12-month assessment point only: there is a significant mean reduction of the total MADRS scores between T0 and 6 month (MADRS_T0_: 34.9 ± 9.9, MADRS_T1_: 11.7 ± 9.5; *p <* 0.001); no differences emerged between 6 and 12-month follow-up (MADRS_T2_: 11.6 ± 9.9; p: ns). In patients who discontinued ESK-NS between 6 and 12-month a further mean reduction of MADRS emerged at 12 month (mean MADRS_T2_: 5.5 ± 5.7).

In patients who continued ESK-NS until the 12-month assessment, a significant reduction in HAM-A scores was similarly observed (HAM-A_T0_: 34.1 ± 8.4, HAM-A_T1_: 13.8 ± 10.3, HAM-A_T2_: 11.0 ± 9.8; *p <* 0.001).

Among characteristics of patients at 6-month, factors significantly associated to improvement with ESK-NS at 6-months treatment were shorter duration of current MDE (12.1 ± 7.9 *vs.* 21.3 ± 15.0: *p =* 0.003) and less psychiatric comorbidity (35.4% *vs.* 73.3%: *p =* 0.010); moreover, but only with a trend of statistical significance, lower MADRS baseline scores (33.0 ± 10.1 *vs.* 38.4 ± 7.9: *p =* 0.063) and lower baseline HAM-A scores (20.0 ± 11.4 *vs.* 27.4 ± 13.1: *p =* 0.057).

Concerning tolerability of ESK-NS, at least one side effect has been noticed in 71.8% of study subjects at 6-month, but only 2 patients discontinued treatment because of tolerability concerns. The most common side effects reported in the first 6 months of ESK-NS treatment were sedation (31.7%) and dissociation (28.6%), as shown in Fig. (**3**). At 12-month assessment point, 42% of patients who continued ESK-NS reported at least one side effect, with an overall reduction of the most common side effects.

## 4 DISCUSSION

This is the first study taking into account the mid and long-term trajectories of TRD patients treated with esketamine in the real-world setting of Italian mental health centers. The aims of the study were to evaluate the effectiveness and tolerability of ESK-NS in real-world TRD patients and to compare the characteristics of subjects who continued ESK-NS and those who did not.

The characteristics of the study sample are in line with the population of patients suffering from TRD treated in Italian mental health services, with recurrent MDD, long duration of current MDE, polypharmacy with more than two antidepressants and/or combination of AD with antipsychotics and/or mood stabilizers and with benzodiazepines also [12].

The first remarkable result is the rate of patients significantly improved at 6-month (76.2%). In other words, 3 out of 4 patients with TRD benefit from ESK-NS within 6 months. This rate should be compared with the response/remission rate of 26.5% after 6 months in TRD patients treated with classical pharmacological approaches [3]. Based on these results, it seems that 50% more patients with TRD can improve if treated with ESK-NS plus an oral AD (SSRI or SNRI) compared to pre-esketamine era. Moreover, the rate of 76.2% found in this study is in line with the 68% rate of response/remission at a 3-month time point in the first REAL-ESK study [9]. The increase in response rates up to 6 months is an extremely noteworthy result, and it is possible that it is at least partially attributable to the multiple effects that ESK-NS has on brain neurotrophism, involving the GSK-3/mTOR pathways and the modulation of BDNF, which often result in effects observable only over time.

In our study, only 7.7% of the total sample stopped esketamine within 6 months because of lack of efficacy and only 2.6% because of adverse events, in line with the safety results at 3 months (2.58% of patients with adverse events leading to discontinuation) [9].

The second main result is the high rate of discontinuation between 6 and 12-month time points: this is due to multiple reasons. First of all, many patients (N=26, 54% of responders/remitters at 6-month) have ESK-NS stopped because of the clinical improvement, as it should be done after six months of treatment; additionally, in clinical practice, the tendency to discontinue the medication after achieving a prolonged treatment response is also due to cost considerations or, at times, unavailability of the medication. Indeed, in these subjects, we noticed a further reduction in mean MADRS scores from 6-month to 12-month assessment points, probably due to the early symptomatic remission. Others stopped ESK-NS due to lack of improvement (N=2, 13% of non-responders at 6-month) and only one patient because of side effects (2.4%). Among the 15 non-responders at 6 months, 4 become responders/remitters at 12-month follow-up, confirming the impression that in a subgroup of patients, ESK-NS shows effectiveness only if the treatment continues for months.

As stated before, the limited sample size of our study allowed analysis of the characteristics of responders *vs.* non-responders patients only at 6 months. Our findings indicate that patients who improved with ESK-NS treatment had a shorter duration of current MDE, lower MADRS and HAM-A baseline scores and fewer comorbid psychiatric disorders. These findings are consistent with data from post-hoc analyses of RCTs showing that attainment of response and remission in patients treated with ESK-NS was more likely in patients who were employed, without significant anxiety at baseline, and who experienced a reduction in CGI-S score at day 8 [13]. Even in a recent study of REAL-ESK group using the machine-learing approach in order to predict the outcome of patients in the acute phase of treatment with esketamine, benzodiazepine usage and depression severity were linked to delayed responses [14]. Indeed, in patients who keep taking esketamine for 12 months, HAM-A scores continue to decrease (even between 6 and 12 months), showing an independent efficacy of esketamine on anxiety symptoms. In other words, the severity of depression and anxiety in terms of intensity of symptoms, duration of the episode and impact on patients’ functioning seems to be negatively related to the likelihood of improvement with ESK-NS, particularly in a short-term perspective.

For what concerns the tolerability profile, sedation and dissociation are the most common side effects in our sample, followed by hypertension and nausea and all side effects tend to reduce over time (from 6 to 12-months assessment point), in line with previous findings. On the basis of the routine clinical practice with ESK-NS and in line with insights coming from RCTs, this compound is basically safe in patients correctly selected and the severity of potential adverse events tends to decrease during subsequent sessions. In other words, no new adverse events usually appear in the mid or long-term run. Therefore, our results highlight that side effects from ESK-NS tend to decrease over time, thereby confirming that the treatment is widely tolerable even for maintenance.

The interpretation of the study results is limited by several factors: first of all, the retrospective observational design may have led to underestimating selection bias, but it also reflects the real-world setting of patients with TRD, enhancing the applicability of the results. Additionally, due to the design of the study, we are unable to provide interim data on the efficacy of the treatment before six months or between six and twelve months. Another limitation is the smaller sample size, which is also due to the exclusion of patients lost to follow-up or with missing data at 6 and 12-month follow-up. Lastly, 12-month is still a limited observational period for what concerns the long-term outcomes of ESK-NS, but today, these are the first observational data on real-world patients in the maintenance phase.

## CONCLUSION

In conclusion, our data support the effectiveness and safety of esketamine in the mid and long-term run. The late clinical response (after 6 months from the beginning of the treatment) of a subgroup of patients represents a novel finding, as well as the further improvement of depressive symptoms in those who stopped ESK-NS due to the clinical efficacy.

Our findings need to be confirmed in larger samples and longer observation periods. Analyses on the characteristics of patients with or without depressive relapses after esketamine discontinuation should also be performed.

## AUTHORS’ CONTRIBUTIONS

The authors confirm their contribution to the paper as follows: study conception and design: GR, MDN, PDF, MC, BMD, AV, GDL, MP, GM, GMa; data collection: GR, G d’A, SB, MDN, IA, MM, VM, MO, SDM, GDS, PDF, BMD; data analysis and interpretation: GR, G d’A, SB, MDN, IA, MM, VM, MO, SDM, GDS, PDF, BMD; writing the paper: GR, G d’A, MP, GM, GMa. All authors reviewed the results and approved the final version of the manuscript.

## Figures and Tables

**Fig. (2):**
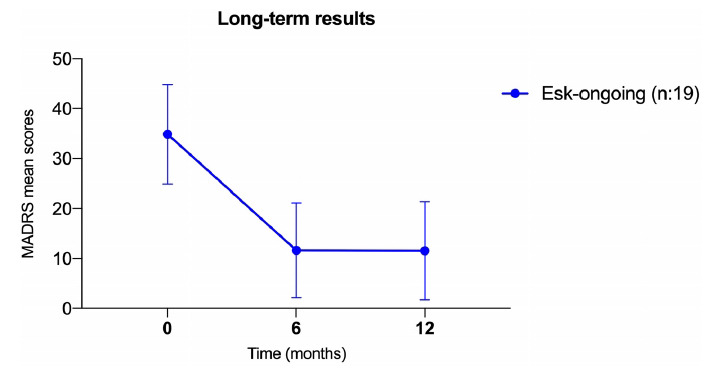
MADRS mean variations at 6 and 12 months.

**Fig. (3):**
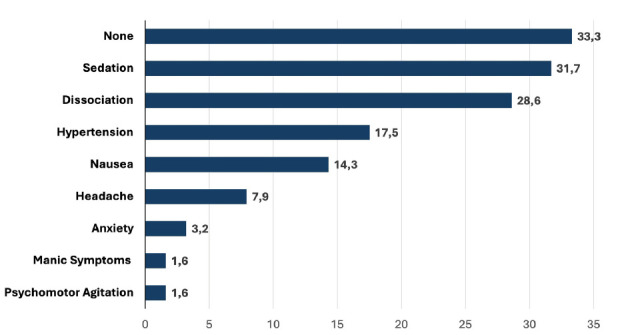
Side effects at 6-month follow-up (% of patients).

**Table 1:** Baseline demographic and clinical characteristics of the sample.

**Parameters**	*N*=78
**Age,** years (mean ± SD)	51.8 ± 12.6
**Sex,***n* (%) Male Female	35 (44.9) 43 (55.1)
**Marital status,***n* (%): Single Married Divorced Widowed	23 (29.5) 42 (53.8) 9 (11.5) 4 (5.1)
**Educational level,** years (mean ± SD)	13.8 ± 3.2
**Occupation,***n* (%) Employed Unemployed Retired Student	42 (53.8) 21 (26.9) 13 (16.7) 2 (2.6)
**Age at onset of MDD,** years (mean ± SD)	32.8 ± 13.1
**Current episode length,** months (mean ± SD)	15.6 ± 11.5
**Number of previous episodes,** (mean ± SD)	5.2 ± 10.6
**Suicide attempts lifetime,***n* (%) Yes No	19 (24.4) 59 (75.6)
**Psychiatric comorbidities,***n* (%) Yes No	37 (47.4) 41 (52.6)
**Number of previous adequate antidepressant trials that proved to be ineffective,** (mean ± SD)	3.3 ± 1.8
**Ongoing pharmacological treatment,***n* (%) SSRI SNRI Other AD Antipsychotics Benzodiazepine Mood Stabilizers	38 (48.7) 30 (38.5) 36 (46.2) 36 (46.2) 38 (48.7) 45 (57.7)
**Non pharmacological treatments,***n* (%) Yes No	16 (20.5) 62 (79.5)
**MADRS scores,** (mean ± SD)	34.9 ± 9.9
**HAM-A scores,** (mean ± SD)	24.1 ± 12.4

**Abbreviations**: MDD: Major Depressive Disorder; MADRS: Montgomery-Asberg Depression rating scales; HAM-A: Hamilton anxiety rating scale; SSRI: Selective serotonin reuptake inhibitor; SNRI: Serotonin and Norepinephrine Reuptake Inhibitor.

**Table 2:** Causes of treatment discontinuation.

**(2a) Between Baseline and Six Months**		
**Stop Treatment (n:15)**	**N**	**%**
Low effectiveness	6	40.0
Clinical remission	1	6.7
Adverse Events	1	13.3
Unknown/Organizing problems	6	40.0
**(2b) Between Six and Twelve Months**		
**Stop Treatment (n:44)**	**N**	**%**
Unknown/Dropouts	13	29.5
Clinical remission	28	63.6
Adverse Events	1	2.4
Relapse	2	4.5
Total	44	100

## Data Availability

All data generated or analysed during this study are included in this published article.

## LIST OF ABBREVIATIONS

AD: Antidepressant
ANOVA: Analysis of Variance
ESK-NS: Esketamine Nasal Spray
HAM-A: Hamilton Anxiety Rating Scale
MADRS: Montgomery-asberg Depression Rating Scale
MDD: Major Depressive Disorder
MDE: Major Depressive Episode
SD: Standard Deviation
TRD: Treatment Resistant Depression

## STANDARDS OF REPORTING

STROBE guidelines were followed.

## References

[1] (2013). European Medicines Agency Clinical investigation of medicinal products in the treatment of depression - Scientific guideline.. https://www.ema.europa.eu/en/clinical-investigation-medicinal-products-treatment-depression-scientific-guideline.

[2] McIntyre R.S., Alsuwaidan M., Baune B.T., Berk M., Demyttenaere K., Goldberg J.F., Gorwood P., Ho R., Kasper S., Kennedy S.H., Ly-Uson J., Mansur R.B., McAllister-Williams R.H., Murrough J.W., Nemeroff C.B., Nierenberg A.A., Rosenblat J.D., Sanacora G., Schatzberg A.F., Shelton R., Stahl S.M., Trivedi M.H., Vieta E., Vinberg M., Williams N., Young A.H., Maj M. (2023). Treatment-resistant depression: Definition, prevalence, detection, management, and investigational interventions.. World Psychiatry.

[3] Heerlein K., Perugi G., Otte C., Frodl T., Degraeve G., Hagedoorn W., Oliveira-Maia A.J., Perez Sola V., Rathod S., Rosso G., Sierra P., Malynn S., Morrens J., Verrijcken C., Gonzalez B., Young A.H. (2021). Real-world evidence from a European cohort study of patients with treatment resistant depression: Treatment patterns and clinical outcomes.. J. Affect. Disord..

[4] Heerlein K., Young A.H., Otte C., Frodl T., Degraeve G., Hagedoorn W., Oliveira-Maia A.J., Perez S.V., Rathod S., Rosso G., Sierra P., Morrens J., Van Dooren G., Gali Y., Perugi G. (2021). Real-world evidence from a European cohort study of patients with treatment resistant depression: Baseline patient characteristics.. J. Affect. Disord..

[5] Heerlein K., De Giorgi S., Degraeve G., Frodl T., Hagedoorn W., Oliveira-Maia A.J. (2022). Real-world evidence from a European cohort study of patients with treatment resistant depression: Healthcare resource utilization.. J. Affect. Disord..

[6] Popova V., Daly E.J., Trivedi M., Cooper K., Lane R., Lim P., Mazzucco C., Hough D., Thase M.E., Shelton R.C., Molero P., Vieta E., Bajbouj M., Manji H., Drevets W.C., Singh J.B. (2019). Efficacy and safety of flexibly dosed esketamine nasal spray combined with a newly initiated oral antidepressant in treatment-resistant Depression: A randomized double-blind active-controlled study.. Am. J. Psychiatry.

[7] Reif A., Bitter I., Buyze J., Cebulla K., Frey R., Fu D.J., Ito T., Kambarov Y., Llorca P.M., Oliveira-Maia A.J., Messer T., Mulhern-Haughey S., Rive B., von Holt C., Young A.H., Godinov Y. (2023). Esketamine nasal spray versus quetiapine for treatment-resistant depression.. N. Engl. J. Med..

[8] Samalin L., Rothärmel M., Mekaoui L., Gaudré-Wattinne E., Codet M.A., Bouju S., Sauvaget A. (2022). Esketamine nasal spray in patients with treatment-resistant depression: The real-world experience in the French cohort early-access programme.. Int. J. Psychiatry Clin. Pract..

[9] Martinotti G., Vita A., Fagiolini A., Maina G., Bertolino A., Dell’Osso B., Siracusano A., Clerici M., Bellomo A., Sani G., d’Andrea G., Chiaie R.D., Conca A., Barlati S., Di Lorenzo G., De Fazio P., De Filippis S., Nicolò G., Rosso G., Valchera A., Nucifora D., Di Mauro S., Bassetti R., Martiadis V., Olivola M., Belletti S., Andriola I., Di Nicola M., Pettorruso M., McIntyre R.S., di Giannantonio M. (2022). Real-world experience of esketamine use to manage treatment-resistant depression: A multicentric study on safety and effectiveness (REAL-ESK study).. J. Affect. Disord..

[10] Daly E.J., Trivedi M.H., Janik A., Li H., Zhang Y., Li X., Lane R., Lim P., Duca A.R., Hough D., Thase M.E., Zajecka J., Winokur A., Divacka I., Fagiolini A., Cubala W.J., Bitter I., Blier P., Shelton R.C., Molero P., Manji H., Drevets W.C., Singh J.B. (2019). Efficacy of esketamine nasal spray plus oral antidepressant treatment for relapse prevention in patients with treatment-resistant depression.. JAMA Psychiatry.

[11] Sforzini L., Worrell C., Kose M., Anderson I.M., Aouizerate B., Arolt V., Bauer M., Baune B.T., Blier P., Cleare A.J., Cowen P.J., Dinan T.G., Fagiolini A., Ferrier I.N., Hegerl U., Krystal A.D., Leboyer M., McAllister-Williams R.H., McIntyre R.S., Meyer-Lindenberg A., Miller A.H., Nemeroff C.B., Normann C., Nutt D., Pallanti S., Pani L., Penninx B.W.J.H., Schatzberg A.F., Shelton R.C., Yatham L.N., Young A.H., Zahn R., Aislaitner G., Butlen-Ducuing F., Fletcher C., Haberkamp M., Laughren T., Mäntylä F.L., Schruers K., Thomson A., Arteaga-Henríquez G., Benedetti F., Cash-Gibson L., Chae W.R., De Smedt H., Gold S.M., Hoogendijk W.J.G., Mondragón V.J., Maron E., Martynowicz J., Melloni E., Otte C., Perez-Fuentes G., Poletti S., Schmidt M.E., van de Ketterij E., Woo K., Flossbach Y., Ramos-Quiroga J.A., Savitz A.J., Pariante C.M. (2022). A Delphi-method-based consensus guideline for definition of treatment-resistant depression for clinical trials.. Mol. Psychiatry.

[12] Perugi G., Calò P., De Filippis S., Rosso G., Vita A., Adami M., Ascione G., Morrens J., Delmonte D. (2021). Clinical features and outcomes of 124 Italian patients with treatment resistant depression: A real-world, prospective study.. Front. Psychiatry.

[13] Turkoz I., Nelson J.C., Wilkinson S.T., Borentain S., Macaluso M., Trivedi M.H., Williamson D., Sheehan J.J., Salvadore G., Singh J., Daly E. (2023). Predictors of response and remission in patients with treatment-resistant depression: A post hoc pooled analysis of two acute trials of esketamine nasal spray.. Psychiatry Res..

[14] Pettorruso M., Guidotti R., d’Andrea G., De Risio L., D’Andrea A., Chiappini S., Carullo R., Barlati S., Zanardi R., Rosso G., De Filippis S., Di Nicola M., Andriola I., Marcatili M., Nicolò G., Martiadis V., Bassetti R., Nucifora D., De Fazio P., Rosenblat J.D., Clerici M., Maria Dell’Osso B., Vita A., Marzetti L., Sensi S.L., Di Lorenzo G., McIntyre R.S., Martinotti G. (2023). Predicting outcome with intranasal esketamine treatment: A machine-learning, three-month study in treatment-resistant depression (ESK-LEARNING).. Psychiatry Res..

